# Usefulness of micro-arrays technology in ethiological diagnosis of eosinophilic esophagitis

**DOI:** 10.1186/2045-7022-3-S3-P42

**Published:** 2013-07-25

**Authors:** A Armentia, J Barrios, B Martin, A Sanchez, N Alcalde, P Fernandez-Orcajo, S Martin

**Affiliations:** 1 Allergy Unit, Hospital Universitario Rio Hortega, Valladolid, Spain; 2 Gastroenterology Service, Hospital Universitario Rio Hortega, Valladolid, Spain; 3 Paediatrics Service, Hospital Universitario Rio Hortega, Valladolid, Spain

## Background

Eosinophilic esophagitis (EoE) is an immune-mediated disease of the esophagus characterized by symptoms related to esophageal disfunction and histollogically by an eosinophil-predominat inflammation. Multiple therapies have been suggested to be helpful in EoE including endoscopic dilation, medical therapy and withdrawal dietary but, the election of what food should be excluded is very difficult.

Component-resolved diagnosis and microarray technology have been recently introduced into clinical allergy practice, and may be particularly useful in food-sensitized allergic patients.

We used microarrays methods in EoE to find specific food proteins or epitopes possibly implicated in this disease.

## Methods

We studied 42 patients suffering from EoE diagnosed by clinical symptoms and endoscopic biopsy esophageal with > 15 eosinophils/high-powered field. Microarray technique (Thermofisher scientific), including detection of 112 allergens were performed in these 42 patients and in 50 allergic controls with pollen sensitization but without digestive symptoms.

## Results

Only 7 of the 42 patients that suffered from EoE did not present any allergen sensitization. All control patients presented sensitization to different pollen allergens without any predominant allergen. Nevertheless, among the 35 patients with EoE and with response to any allergen, the predominant were nCyn d 1 (*Cynodon dactylon* or Bermuda grass pollen) 59.5%, and the following allergens: Lipid transfer proteins (LTPs) from peach (26.2%), hazelnut 26.1% and mugwort 23.8%. Profilins were positive in 9.5% of the patients. Among nuts, allergens from hazelnut and walnut (21.4%) were the most important. Other food allergens as Anisakis, egg or milk, only were positive in 9.5%, 2.3 and 4.7% respectively.

## Conclusion

High sensitization to vegetable allergens is relevant among patients with EoE. The most important implicated allergens are LTPs (usually associated to severe allergic response) from nuts and fruits and antigen 1 from Cynodon dactylon. Our patients are being treated with exclusion of the implicated food and pollen specific immunotherapy with preliminary favourable results.

## Disclosure of interest

None declared.

